# Richmond Academy of Medicine and Surgery

**Published:** 1895-04

**Authors:** 


### *SOCIETY REPORTS.*

---

### RICHMOND ACADEMY OF MEDICINE AND SURGERY.

Richmond, Va., February 12, 1895.

Dr. Landon B. Edwards was the leader in the subject for the evening—"The Therapeutics of Gout, Uric Acid Diathesis, and Gravel"—the whole subject of gout, however, being open for discussion. Poor man's gout is due to half-masticated food washed down by draughts of heavy beer and to lack of exercise. Gout may also be caused by lead. An excess of highly seasoned food, in fact, excess of any kind, directly predisposes to it. The disease implicates oftenest the nervous systems. Of course, it is directly due to the increased formation or diminished elimination of uric acid. There are strong reasons for attributing to the liver the chief part in the formation of urea. So long as this organ is active the kidneys carry it off; but if, for any reason, the kidneys and liver do not perform their duties, retention of uric acid occurs and the results may be manifested in gout, regular or irregular, with its attendant symptoms; in the formation of tophi or calculi, renal or vesical.

*Treatment.*—Children of gouty parents must do more than live temperately, both as regards food and drink. They must take plenty of exercise. An indication for treatment is to prevent catarrhal conditions of the urinary tract. Use milk and an abundance of alkaline waters. In the growing child, use fish, eggs, cereals, etc. Avoid overeating, especially highly seasoned food and dark meats. Clothing must be suited to the season.

*Medical.*—In the examination of the urine, extractives should be looked for as well as uric acid and albumins. For constipation, nothing is better than cascara. In the beginning of the attack, use moderate doses, twelve to fifteen drops of the wine or tincture of colchicum seed, night and morning. Aconite, in drop doses, may be combined with it. The action of colchicum varies according to idiosyncrasy, some people being able to take a teaspoonful without discomfort, while small doses, in others, produce fatal effect; and in any event, the vomiting, which may occur, is objectionable. Salicylate of sodium is a specific in this disease and more. It assists in the elimination of uric acid first, and then prevents its further formation. It shows its virtue in preventing both acute and chronic gout.

During the intervals no one medicine excels iodide of potassium in small doses and at long intervals. For neurotic troubles, during the intervals, use dilute solutions of phosphate of sodium. Especial value is attributed to the use of the lithium salts. I have confidence in the lithia waters, especially for the results of gout. I have no doubt that calculi can be reduced in the system and washed out by the urine. A case occurring in my practice is in point. The man claimed descent from noblemen. The father was a gourmand and beer-drinker; and his children had gout and renal calculus. In one child the latter was pronounced. Operation was declined. The diet was restricted. Buffalo Lithia water prescribed, and in a few days the urine was better, calculi passed, all symptoms disappeared, and, so far as I know, never reappeared. The effect of lithium and allied salts is to alkalinize the blood. It is proved by authorities that waters containing even but traces of lithium possess solvent action on uric acid gravel and tophi. In one report seventy-three per cent. of the cases collected showed solution of stone, and in the balance crushing was facilitated.

### DISCUSSION.

DR. HUGH M. TAYLOR.—Dr. Edwards said, in order to be free of gout, one must be poor. Now, according to authorities, the rich man has gout, but his child is free from stone; the poor man is free, but his child has stone. The reason is the rich man's child has his food carefully selected, is given opportunities for taking all the exercise he wishes, and has no anxiety whatever. The poor man, although he eats indigestible, proteid food and drinks heavy beer, is obliged to labor for his living. His child is scantily clothed, lives in unsanitary dwellings, and his food is not alone scanty, but indigestible. I have often been struck with the fact that vesical calculus is uncommon in the negro, and can recall but one case in the whole race in my practice. The explanation as to the adult is the same as in the case of the poor man, but I cannot understand why the child is exempt.

DR. JACOB MICHAUX.—I am struck by Dr. Edwards's statement as to the small quantities of salts acting favorably. If the doses are full, the stomach revolts, and especially is this true of the salts of lithium. We forget that we are injuring the digestive functions in giving large doses of alkali. The success of the mineral waters is due to the small amounts of the salts they contain, and to the fact that patients take them instead of water.

DR. J. S. WELLFORD.—In the uric acid diathesis there isn't sufficient metabolism. I contend that rheumatism and gout are but different phases of the same thing. I don't believe in the lactic acid theory, and I hold that lithemia is a blood poisoning due to uric acid, the same being true in the sequelæ of scarlet fever and in dysmenorrhea, most particularly in the latter if the patient has gouty parents. In one case in my practice, the patient was free during the reproductive period; but as soon as the menopause came on, gout manifested itself. Whenever a person is making more nitrogenous matter than he can dispose of, gouty symptoms occur. The skin plays an important part in elimination, as do the liver, intestines, etc. If, for any reason, they do not act, extra labor is thrown on the kidneys and susceptibility to gout occurs.

My theory as to the cause of vascular and cardiac complications and sequelæ is, the lining membrane of the left side of the heart and of the arteries is intended for alkali or fluid, that of the right side and veins for acid. As soon as the blood for any reason becomes acid, the arteries and left side of the heart are affected and we have arteritis, cardiac palpitation, angina, and valvular trouble. We never have phlebitis in gout. As to the joints, they have less circulation than other parts of the body and the blood is less alkaline. They are therefore disposed to stagnation and deposit.

Anything producing increased quantities of nitrogen or lessened metabolism creates gout. After the food is taken in, digested, assimilated, it is rendered effete and thrown into the venous system. Prior to and during this time, it undergoes chemical changes and leucomaines are formed. Urea is the first of these, then uric acid, and if indigestion occur, otalic acid.

A number of people have gout because they do not take enough water, the uric acid being too concentrated.

In the treatment of gout, I am of the opinion that mischief may be done in trying to relieve too suddenly by stopping elimination, and confirmed gout may result. Oil of peppermint is the best local application I have tried. It is soothing and aids in the solution of the acid. In constitutional treatment, don't use too much opium, for the reason given above; it prevents elimination of the acid. Of the alkalies the salicylates, to a certain extent, are the best, especially that of potash. I believe the mineral waters subserve a useful purpose by flushing, and am satisfied that I have saved myself frequently from attacks of gout by the free ingestion of water. Theoretically lithium ought to be the best article in gout; but, in my experience, I have not found it of half the value of potash, the urate of the latter being more soluble. All of the potash salts are diuretic, soda, cholagogic. The main treatment should be in diet and exercise. The reason we have so much more gout now than previously, is to be found in the use of the street-cars, and in the case of physicians' buggies. The diet must be regulated by the individual. No hard and fast lives can be set down. Let it be liberal. Diminish the nitrogenous food and liquors, or if the latter must be used, give good whisky. Use carbohydrates.

DR. MARK W. PEYSER referred to the connection between the leucocytoses and uric acid. The acid is related closely to the extractives xanthin, sarkin, quanine, adenine, etc. Spleen nuclein does not contain uric acid nor these, but it does contain the mother-substance from which they may be made, the acid being formed in the presence of an oxidizing substance, the others being formed in its absence. The nuclein is derived from the colorless corpuscles, and the amount of urea and uric acid formed is a measurer of nuclein metabolism. Any condition, then, which produces leucocytosis increases the production of uric acid. Hence, we see it when a large amount of proteid food is taken in, in leucocythemia phosphorus poisoning, acute febrile diseases (especially pneumonia) in infants, pernicious anemia, etc.

The doctor referred to the intimate relation existing between gout and diabetes mellitus, and Packard in Hare's System of Therapeutics quoted in regard to it. This authority cites table given by Charcot, showing that in three generations of one family the first contained a gouty individual; the second, four gouty and four diabetics; and the third, one gouty.

DR. E. C. LEVY mentioned a case illustrating the effect of diminished ingestion of liquids, which was speedily relieved by the free use of water. The reason that carbohydrates are not advised, said the doctor, is because of the large amount of oxygen required for their oxidation. He has had good results in the use of Piperazine.

DR. EDWARDS, in closing the discussion, said that, beginning with 1875, we could trace developing cases of gout. From 1860 to 1875, it was rare to hear of it, because of the struggle necessary for livelihood. Piperazine has been tried and found wanting, clinically. The chemist in his laboratory found it perfect. Medical treatment should be tentative, drugs being given in small doses. Large doses of colchicum cause stomach troubles, and are not inapt to produce death. I am, he said, in accord with Dr. Wellford as to relieving too suddenly. The salicylates act as solvents, and not as the physiologists wished them to do. They are, by far, the best remedies in the production of a cure. As to diet, avoid tomatoes on account of the oxalic acid contained in them. The presence of uric acid in the blood greater than 1 to 6,000 will cause its precipitation and gout; 1 to 7,000 gives rise to the formation of crystals and the manifestation of gouty symptoms. Beyond this amount, it has no effect. Dr. Wellford spoke of uric acid as a leucomaine. According to Roberts, hypodermic injection of it does not produce gout, and he (Roberts) says it is the mechanical action that gives rise to disease.

---

RICHMOND, VA., February 26, 1895.

DR. HUGH MCGUIRE read a paper on Lymphadenoma, commonly known as elephantiasis, the former better expressing its pathology. The nature of the disease, its causes, and symptoms were gone into, and then the treatment was taken up.

If the disease is seen in its early stage and proper treatment started, it may be relieved or, at least, held in check; but, if the trouble has become established, under our present methods little can be accomplished without the aid of the knife. During the inflammatory attacks, the patient should be put to bed, hot or cold applications and the usual remedies used. If the fever is high, anti-thermal agents should be employed. Tonics, such as quinine, iron, cod-liver oil, and the mineral acids can be profitably administered; but perhaps the most valuable medicament is iodide of potash. If the patient be living in a tropical country, he should, of course, be advised to move. After the inflammatory attack has subsided, inunctions of iodine and mercurial ointment should be used to soften the skin and promote absorption. Much good has been done by firmly bandaging the parts with a woolen or, better still, a rubber bandage. Lately, strong galvanic currents have been highly recommended. Ligation of the main artery of the limb and excision of a section of the sciatic nerve have been tried, and, occasionally do good; but neither can be relied upon. In advanced cases of lymphadenoma of the genitals, the affected parts should be amputated. Recent authorities also advise removal of large wedges of the affected tissue when the legs are involved. If the patient's condition does not allow removal of all the growth at one sitting, several operations may be done. In the operations, the most rigid aseptic precautions are necessary, because of the intimate connection this growth has with the lymphatic system.

Before concluding, I would like to report an interesting case of lymphadenoma which has been under my treatment for some weeks. L. C., colored female, aged twenty, has lymphadenoma of the lower limbs, the right more than the left. The greatest measurement of the right calf is 33 inches; thigh, 35 inches. Both legs are eczematous. Seven years ago, the patient suffered from in-growing toenail of the right foot. An eruption started from this, the parts became hot and swollen, and she suffered from severe pain and fever for several days. On an average of once every one or two months, she had acute attacks of the disease, and, after each, the leg has increased in bulk until now it has reached an enormous size. About three years ago, the left leg became involved, and has steadily grown worse. The attacks are more severe in summer, and any unusual amount of work or walking will bring on the trouble, but complete rest of the limb is equally injurious, as there is then an accumulation of lymph in the parts, causing great tension. Any abrasion of the skin is followed by a discharge of lymph, which gives temporary relief. During attacks, she has sharp, shooting pains in the groin and calf, the limbs become stiff, glands swell, and there is high fever. This condition lasts for a day or two, then gradually subsides, leaving the limbs larger and the general health impaired. The legs are now enormous, and locomotion is difficult.

In treating her, I have, at Dr. Hunter McGuire's suggestion, departed from the usual method. Three or four times weekly, I apply, over some of the main lymph channels of the leg, a cup-shaped electrode which contains, one day, a saturated solution of iodide of potash, and, the next, tincture of iodine. A galvanic current of seven or eight milliampères is used for cataphoresis. Whether this treatment will give any permanent relief, I am, as yet, unable to say; but, since it was begun, the calf measurement has been reduced from thirty-four to thirty-three inches, and the patient has passed a longer period without an acute attack than she has known for years. The general health has been improved by tonics, and she is advised to take a moderate amount of exercise. The doctor exhibited photographs of the case.

DISCUSSION.

DR. HUGH M. TAYLOR: I am of the opinion that we do not know enough of the diseases of the lymphatic system. It has an important part in the economy, closely related to that of the veins. The lymphatics are the great sewers; they cast away septic matter. To see how soon they act, watch a septic wound. But, besides this, they convey antiseptic material, and this should teach us the value of using antiseptics, which are absorbed from the surface by the lymph radicles and carried to the deeper parts. As I understand the question, it is due to the blocking of the deeper lymphatics, producing inflammatory troubles, overgrowth, etc., just as obstruction to the veins would cause edema. Elephantiasis may start as a surface injury, spread through the lacunæ and radicles to the deeper vessels and main channels, and even affect the glands, but not necessarily the latter. The glands may be affected in disease without involvement of the vessels, acting as catch-pit for the septic material which has been conveyed to them by the pipes, the lymph vessels. There are two forms of elephantiasis: (1) Spurious, due to obstruction and inflammation of the radicles, first, and then the deeper channels. (2) True, due to a germ found in the tropics and semi-tropics. I cannot see what treatment should be adopted, except to produce absorption of the obstruction.

